# Granular Cell Tumours of the Breast: A Case Series and Literature Review

**DOI:** 10.7759/cureus.87336

**Published:** 2025-07-05

**Authors:** Sofeah Samsuddin, Lester Chee Hao Leong, Cheryl Hui Shan Lim, Chow Yin Wong, Ying Ching Tan

**Affiliations:** 1 Department of Breast Surgery, Singapore General Hospital, Singapore, SGP; 2 Department of Diagnostic Radiology, Singapore General Hospital, Singapore, SGP; 3 Division of Surgery and Surgical Oncology, National Cancer Centre Singapore, Singapore, SGP; 4 Breast Surgery, Singhealth Duke-NUS Breast Centre, Singapore, SGP

**Keywords:** breast, breast tumours, cancer, granular cell tumour, granular cell tumours of the breast

## Abstract

Granular cell tumours (GCTs) are rare tumours that can occur in any part of the body but are commonly observed in the skin, digestive tract, and subcutaneous tissues, with the oral cavity being the most common anatomical location. Breast involvement has been reported in a small proportion of cases and is commonly benign. However, some of these lesions can be malignant. In this study, two cases of benign granular breast cell tumours in women aged 31 and 39 years are highlighted and discussed. This study shows similarities between the clinical and radiological presentation of GCTs with breast malignancies and the associated management difficulties that can be encountered.

## Introduction

Granular cell tumours (GCTs) are rare tumours that were first identified by Abrikossoff in 1926 [1]. They were initially thought to be a form of myoblastoma, but with the aid of immunohistochemical stains and electron microscopy, they are now believed to be of Schwann Cell derivation [1-2]. These tumours can occur in any part of the body, but are commonly observed in the skin and subcutaneous tissue (30% of patients), breast (15% of patients), and digestive tract (5-11% of patients)[1-3].

Breast involvement has been reported in 10-15% of cases of GCTs, with cases reported in males as well [2,4]. Most of these tumours are benign in nature, but 1-2% of these lesions can be malignant, with a poor prognosis, and surgery remains the only curative option [1, 5-6]. These tumours can mimic breast carcinoma both clinically and radiologically, making it difficult to distinguish from breast malignancies. Histopathological examination remains the only method of confirmation [4]. Benign forms of GCTs demonstrate polygonal cells with granular, eosinophilic cytoplasm, small nuclei and may also exhibit mild atypia, although not to the degree of malignant forms. Malignant forms are associated with features such as high mitotic index and pleomorphic cellular tissue. The cells typically stain positive for S100 and CD68 and negative for ER receptors [1,7].

The only recognised treatment for GCT is surgery. Surgical treatment involves complete excision of the neoplasm along with the overhanging mucus and the underlying periosteum (if any) [1]. GCTs are rarely studied tumours, with the largest clinicopathological study involving only 110 patients, with only 56 patients having adequate follow-up [2]. The largest case series of breast GCTs to date consists of 17 patients [8].

## Case presentation

Patient A, a 31-year-old nulliparous, premenopausal female with no personal or family history of any malignancy, presented with a palpable left breast lump for seven months. She did not notice any increase in size, skin, or nipple-areolar complex changes. There was no breast pain or nipple discharge. On examination, patient A had a palpable 1.5 cm mobile lump at the 12 to 1 o’clock position in the left breast, 3 cm from the nipple. Her physical examination was otherwise unremarkable.

Patient B was a 39-year-old nulliparous, premenopausal female with no personal or family history of any malignancy who presented with a palpable right breast lump of two months’ duration without any other associated features. On examination, there was a palpable, hard, 1 cm right breast lump at the 3 o'clock position, 3.5 cm from the nipple. Her physical examination was otherwise unremarkable.

Imaging

Patient A underwent an ultrasound assessment while patient B underwent mammographic and sonographic assessments for imaging work-up. A mammogram was not performed for patient A in view of her young age.

**Figure 1:**
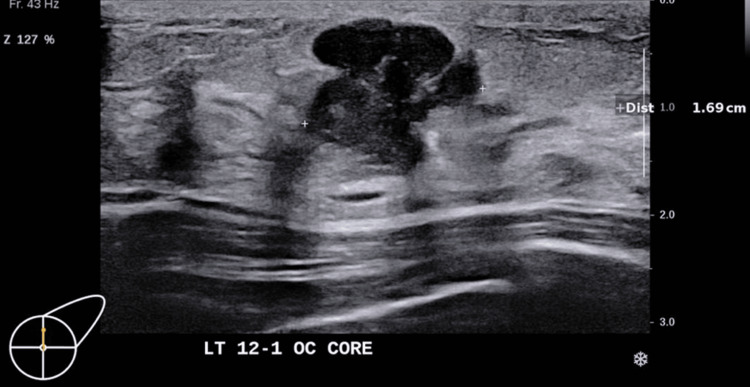
Ultrasound left breast of patient A.

In Patient A, ultrasound assessment showed a 16 mm superficial, highly lobulated but largely well-defined, hypoechoic mass at the 12:30 position (Figure 1). The mass showed mild internal vascularity on Doppler assessment. There were also thin internal hyperechoic internal septations, and no stromal distortion was noted.

**Figure 2:**
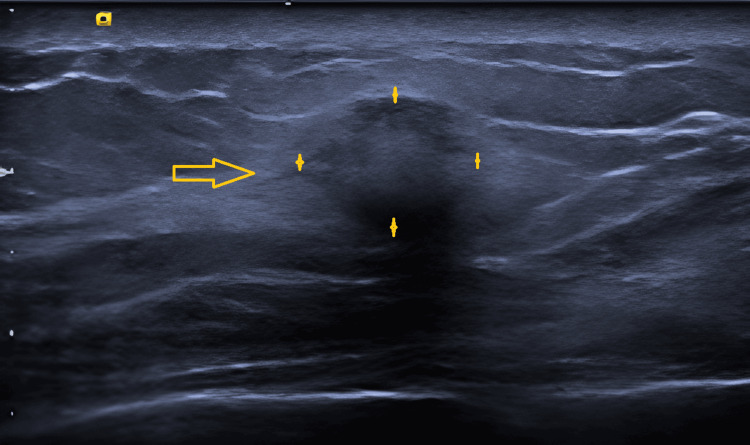
Ultrasound right breast of patient B (arrow indicating lesion as marked between four crosses).

**Figure 3:**
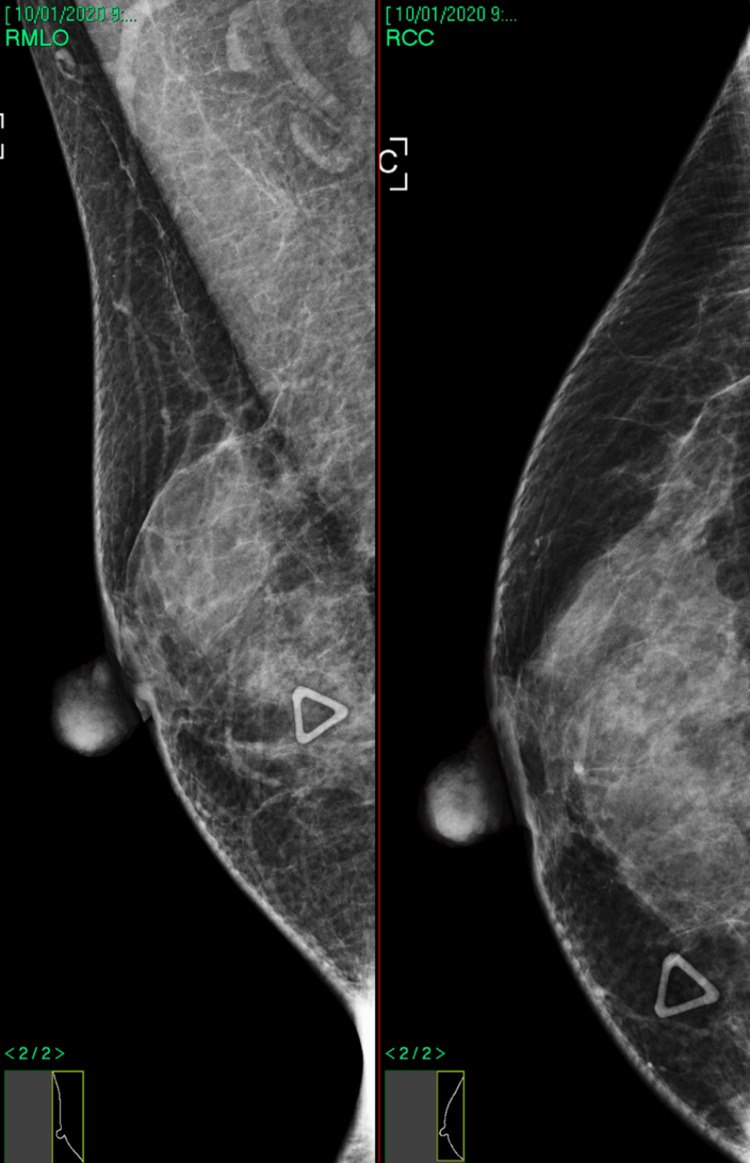
Mammogram image of patient B’s right breast (symptomatic breast).

In patient B, there was an ill-defined, 12 mm rounded mass at the 3 o’clock position. It exhibited increased vascularity, and there was a mix of hyperechoic and hypoechoic areas internally (Figure 2). The lesion was mammographically occult, and there were no associated calcifications or stromal distortion (Figure 3).

No axillary lymphadenopathy was detected in either patient.

Both patients underwent ultrasound-guided core needle biopsy, which obtained two samples of yellow, firm tissue. Patient A subsequently underwent a wide excision in our institution, while patient B sought further treatment elsewhere. 

Histopathology

**Figure 4:**
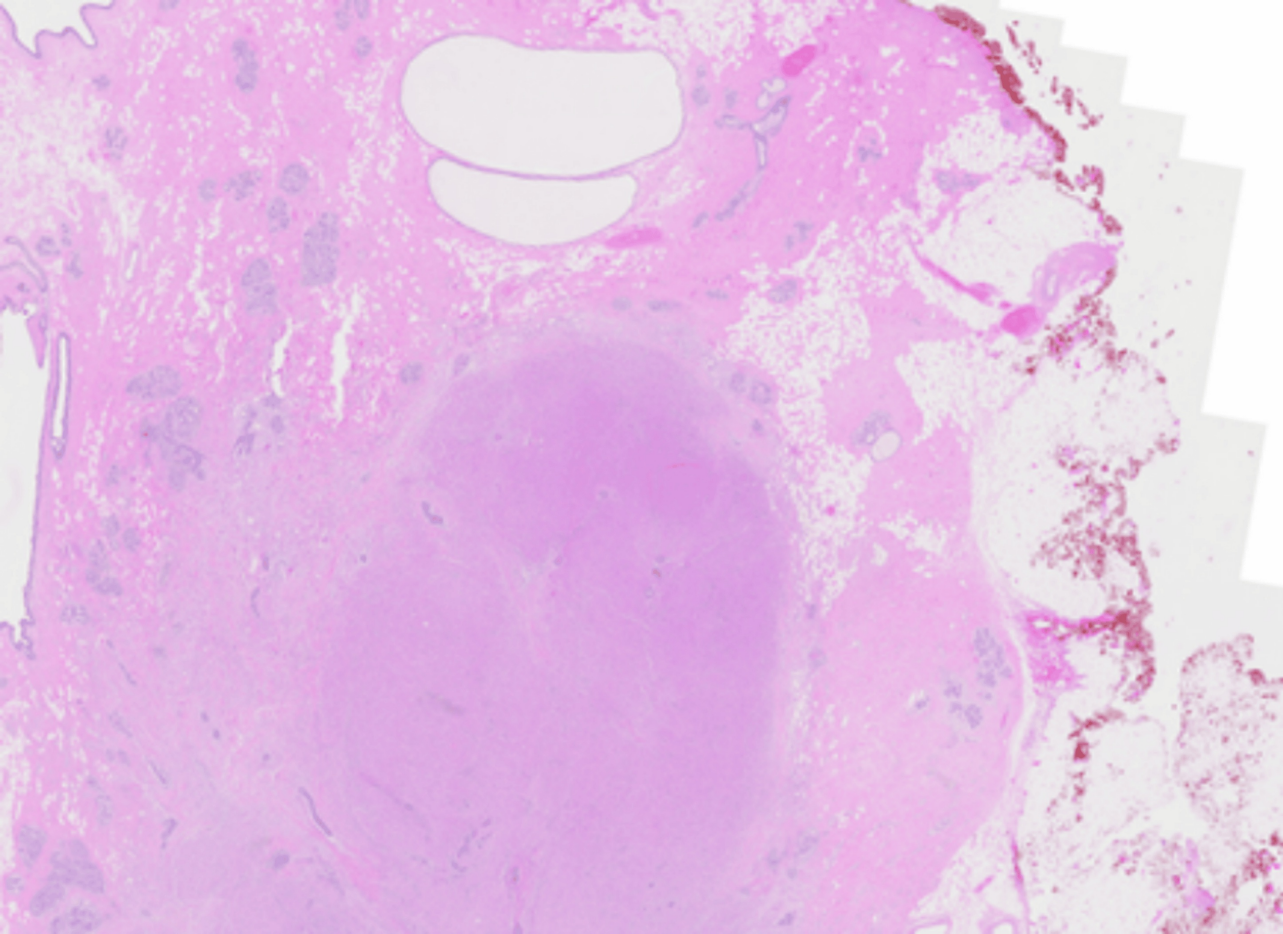
Hematoxylin and eosin (H&E) slide (0.5X magnification): a nodular lesion is seen within the breast parenchyma on low-power examination.

**Figure 5:**
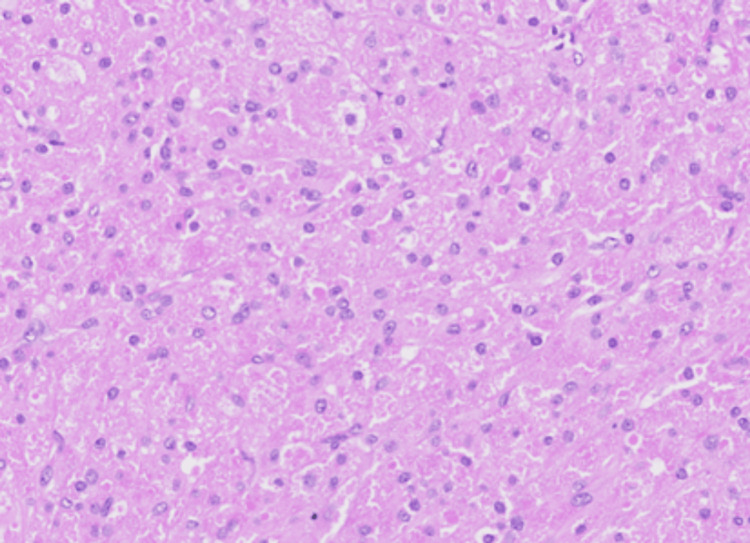
Hematoxylin and eosin (H&E) slide (20X magnification): The nodule is composed of a solid proliferation of tumour cells with abundant granular eosinophilic cytoplasm, featuring round to oval nuclei with distinct nucleoli.

**Figure 6:**
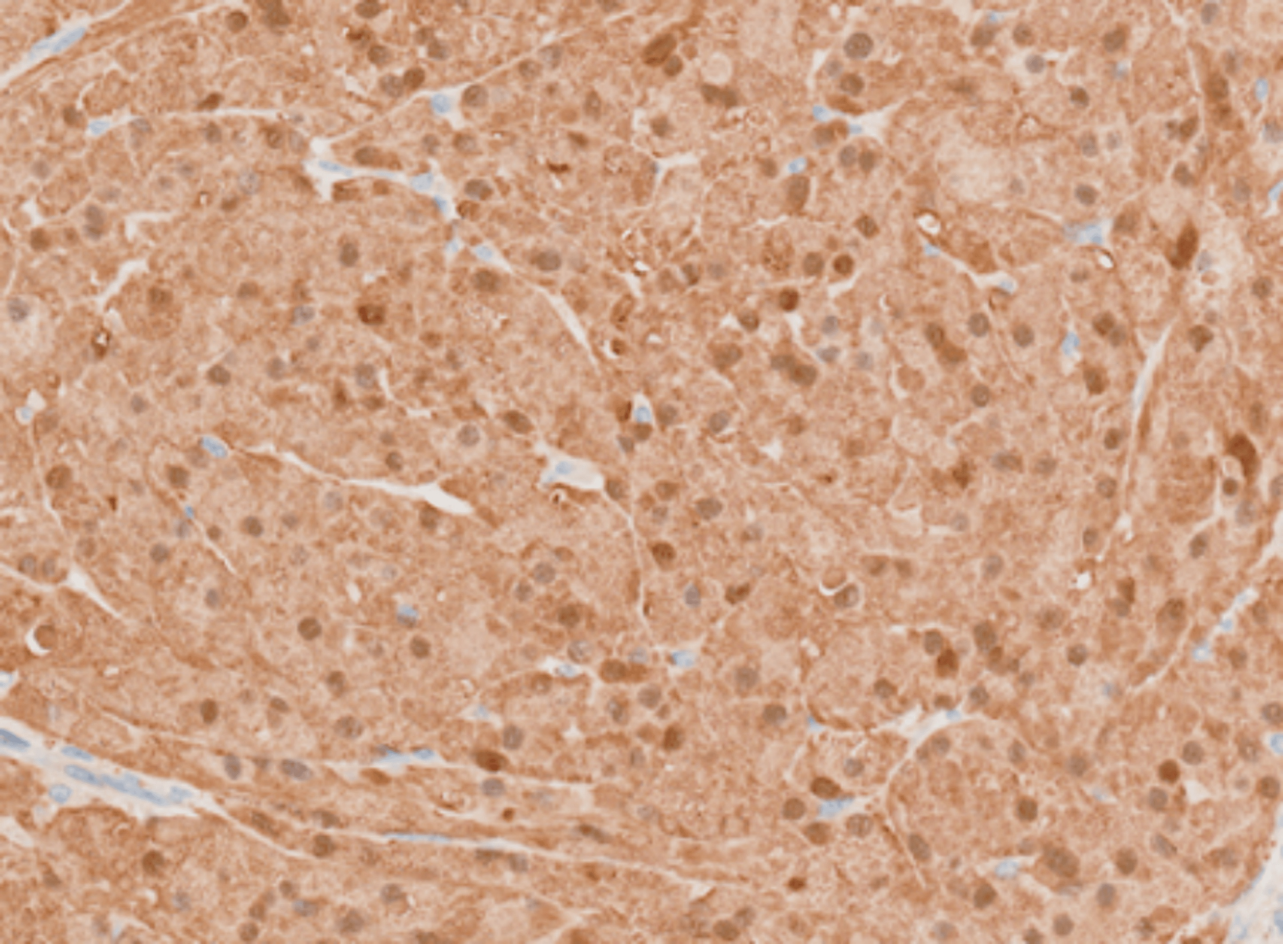
20X magnification: Tumour cells show diffuse immunoreactivity for S100.

**Figure 7:**
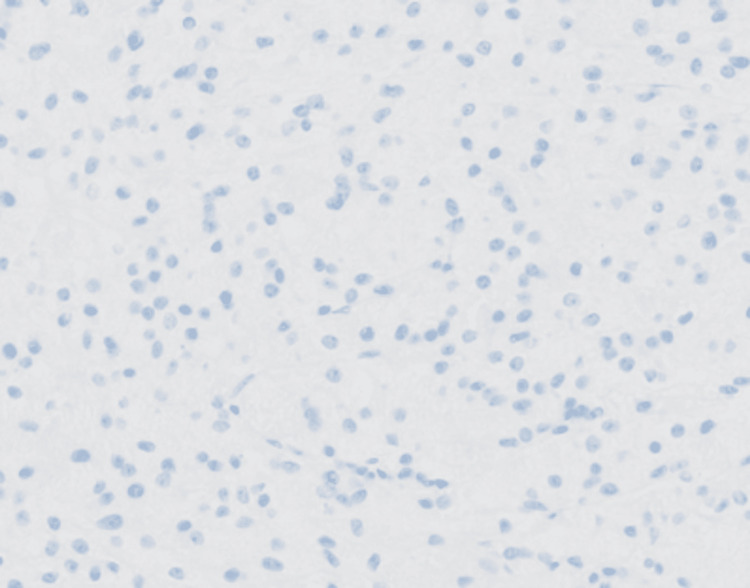
20X magnification: Tumour cells are negative for ER by immunohistochemistry.

In patient A, microscopic sections showed a relatively well-defined nodule composed of a proliferation of lobulated architecture with abundant granular eosinophilic cytoplasm, darker than the surrounding breast tissue, featuring round nuclei with prominent nucleoli. (Figures 4, 5) There were fibrous septations between the lobules, which may correlate with the hyperechoic internal septations seen on sonography. The cells were diffusely positive for S100 (Figure 6), while remaining negative for ER (Figure 7) and p63 (metaplastic breast cancer) and CK14 (myoepithelial or basal cell breast cancer). There were also incidental microscopic foci of atypical ductal hyperplasia less than 1.5 mm in extent, <0.5 mm to the closest inferior margin, clear of other margins. No features of in-situ or invasive carcinoma were seen. Her core biopsy specimen showed similar features. 

In patient B, the core biopsy was found to have infiltrating nests and cords of polygonal, bland cells within a collagenous stroma on core sections. The neoplastic cells showed ill-defined cell borders, abundant granular eosinophilic cytoplasm, and round to oval nuclei with occasional large ones. No mitotic activity was seen. There was scattered chronic inflammation. Few dilated ducts and acini with mixed inflammation were noted both within and at the periphery of the lesion. On immunostaining, the cells were positive for S100, inhibin-alpha, vimentin and CD68. ER and cytokeratin (seen in basal cells of the breast) stains were negative. 

Management

Patient A underwent wide excision of the palpable breast lump in December 2023. In view of a close inferior margin of <0.5mm, the case was discussed at a multidisciplinary board involving a panel of radiologists, pathologists, and breast surgeons to decide further management. While there were no international guidelines regarding a minimum margin, a decision was made for yearly mammogram and ultrasound surveillance for the lesion, as well as for the underlying increased breast cancer risk associated with atypical ductal hyperplasia. The patient was recurrence-free at one year.

Patient B sought further treatment at another institution and was lost to follow-up.

## Discussion

GCTs are uncommon tumours that can arise anywhere in the body, most commonly in the oral cavity, especially the tongue. Breast involvement has been reported in 10-15% of cases of GCTs [2,4]. The histogenesis of GCTs is controversial, but they are currently proposed to be of Schwann cell origin [1,2].

GCTs of the breast have been reported in a wide range of 15-74 years of age [6] and have been reported in both genders [5,9]. However, they most commonly present in premenopausal women with an average age of 32 years [6] and show a slight predominance for those of African American origin [2,8].

GCTs of the breast commonly present with similar findings to those of breast malignancy, making it difficult to differentiate. It usually presents as a painless, hard palpable lump, and they often have features of skin dimpling, tethering, and retraction due to its superficial location [6,10]. It is thought to be derived from the supraclavicular nerve, which innervates the breast skin, hence explaining its commonly identified location in the superficial, upper inner quadrant [10,11]. However, they have been reported in other locations of the breast, such as the two case studies in this report, with 12:30 and 3 o'clock positions. Some cases report tumours fixed to the pectoralis muscle and hence mistaken as malignant breast lesions [10,11]. They are usually unifocal tumours, but multifocal lesions have been reported in 5.4-17.6% of cases [3].

The radiological appearance of various GCT masses can vary significantly. As demonstrated in our series, the margins may appear well-defined; however, if reactive fibrosis is present at the periphery, they may become indistinct. In addition, the masses can exhibit a regular shape, an irregular shape with spicules, or a lobulated appearance with nodular protrusions. However, they do not feature signs of calcifications in any of the case studies [3,12].

On clinical and radiographic examination, it can be impossible to distinguish between GCTs and malignancies of the breast, such as invasive ductal carcinoma. Histopathological diagnosis with core biopsy is a well-established procedure for definitive diagnosis of GCTs [8]. These cells are arranged in nests, clusters, and sheets of polygonal cells with abundant eosinophilic granular cytoplasm from which the tumour derives its name. The granular appearance is due to the cytoplasmic accumulation of lysosomes. Nuclei are small, round and centrally located [1,11]. These tumours do not display mitoses, pleomorphism, cytologic atypia, or necrosis, which indicates the benign nature of most GCTs [10,11]. Accurate diagnosis is often challenging in view of its rare nature and varied appearance. 

There have been rare cases of malignant GCTs (1-2%). The suggested criteria for malignant GCTs include neoplastic necrosis, spindling, vesicular nuclei with large nucleoli and increased mitotic activity, high nuclear to cytoplasmic ratio and nuclear pleomorphism. If three or more criteria are met, the lesion is considered a malignant GCT and is classified as atypical if two of the criteria are met [3,7,8].

The histopathological confirmation of GCTs is often made with immunohistochemical staining. GCTs stain positively with S100 (strong and diffuse), CD68 (weak), and, in some studies, vimentin and inhibin alpha [7]. The strong immunoreaction with S100 protein and its structural appearance resulted in the postulation of GCT as of Schwann cell origin [1,7]. Notably, there have been identified non-Schwann cell subtypes of GCTs, which are S100 negative, and hence the histological origin has yet to be confirmed. They stain negative for estrogen receptors, cytokeratin, and epithelial membrane antigens [7,11].

Gross histopathology of the lesion is of a hard, homogenous, tan, or yellow coloured tumour, most usually well circumscribed. Some case reports show ill-defined borders with infiltration into the pectoralis muscle [10,11,13]. There have not been cases of axillary lymphadenopathy except for GCT that originated in the axillary tail of the breast [8,11].

Wide excision with margins free of tumour is recommended for GCTs of the breast. However, there have not been any well-established margins that are required to prevent recurrence. In a study of 110 patients with granulosa tumours, lesions were incompletely excised in 24 of 56 patients having adequate follow-up, and only five of these 24 patients experienced a local recurrence of tumour. Malignant behaviour was not observed in any of the patients in this study [2].

## Conclusions

GCTs are rare neoplasms that arise from Schwann cells. They present a diagnostic dilemma due to their close clinical and radiological mimicry of breast malignancy. The definitive diagnosis is often made on histology, and tumour excision is often curative. This case study shows the importance of considering GCTs as a probable and alternative diagnosis to breast cancer to avoid erroneous diagnosis of malignancy and unnecessary treatment such as sentinel lymph node biopsy, mastectomy or radiotherapy.
